# A measurement, quantitative identification and estimation method(QINRW) of non-rainfall water component by lysimeter

**DOI:** 10.1016/j.mex.2019.11.012

**Published:** 2019-11-21

**Authors:** Qiang Zhang, Sheng Wang, Ping Yue, Runyuan Wang

**Affiliations:** a Key Laboratory of Arid Climatic Change and Reducing Disaster of Gansu Province, Key Open Laboratory of Arid Climate Change and Disaster Reduction of CMA, Institute of Arid Meteorology, CMA, Lanzhou, China; b Gansu Meteorological Bureau, Lanzhou, China; c Key Laboratory for Semi-Arid Climate Change of the Ministry of Education, College of Atmospheric Sciences, Lanzhou University, Lanzhou, China

**Keywords:** A quantitative identification method for NRW(QINRW), Non-rainfall water(NRW), QINRW(quantitative identification method for NRW), Lysimeter, Land surface water (LSW) balance, Dew, Water vapor adsorption(WVA), Fog

## Abstract

Non-rainfall water (NRW) has an important impact on the ecosystem, especially in arid and semi-arid areas. It is also an important component in the surface water cycle. Currently, there is not any instrument that can directly measure NRW and it can only be estimated by observation data. Presently, there is no standard method available to estimate each constituents of NRW. With some research not distinguishing each component of NRW, this inaccurate methodology will consequently lead to a greater scope for statistical error. Naturally, this compounds the difficulty in evaluating the role of NRW on the ecosystem and land surface water cycle. Therefore, this paper proposes a new methodology for separating NRW components, which is called QINRW(A Quantitative Identification method for NRW). Based on lysimeter data and combined with meteorological data, this method distinguishes the physical properties of each component of NRW. Consequently, the amount of NRW can be obtained. It is also suitable for microlysimeter data to be applied in QINRW.

The advantages of QINRW are three points:

• It is more accurate for excluding the precipitation and dry deposition information from lysimeter data, which was not mentioned in previous studies;
• It can obtain each component of NRW;
• The identification process is more rigorous and clear in theory so far.

## Graphical abstract

**Figure:**
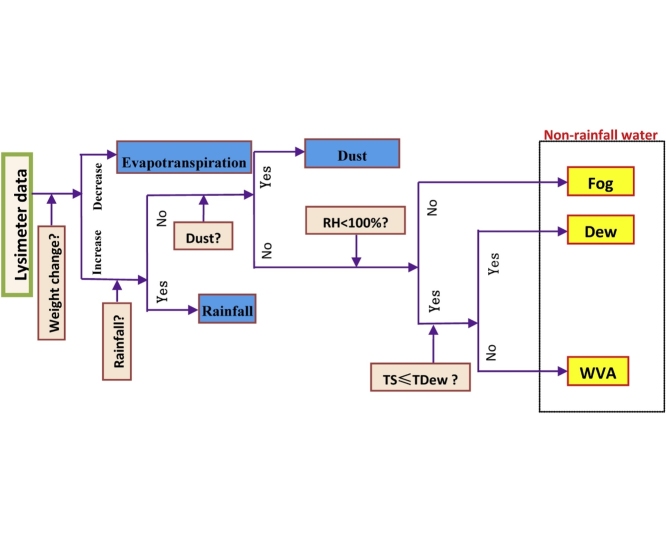

SPECIFICATIONS TABLE

**Table:** 

**Subject Area**	Earth and Planetary Sciences
**More specific subject area**	Land-atmosphere interaction
**Method name**	A quantitative identification method for NRW(QINRW)
**Name and reference of original method**	1. Zhang, Q.,Wang, S.,Zeng, J., 2010. On the non-rainfall water components and their relationship with soil moisture content in arid region. Arid Zone Res.,27,392–400.(in Chinese) 2. Zhang Q., Wang S., Wen X.M., and Li H.Y., 2011: Experimental study of the imbalance of water budget over the Loess Plateau of China. Acta Meteor. Sinica, 25(6), 765–773, doi:10.1007/s13351-011-0607-5.
**Resource availability**	*If applicable, include links to resources necessary to reproduce the method (e.g. data, software, hardware, reagent)*

## *Method details

### Background

**Fig. 1:**
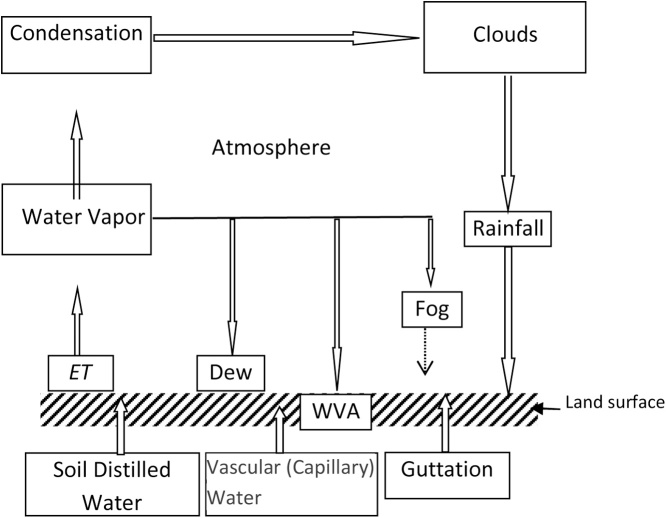
Diagrammatic sketch of the water cycle on the land surface.

Land surface water process refers to those processes including transport, exchange and phase transformation of water vapor occurring between the atmosphere and land surface. Non-rainfall water (NRW) refers to land surface liquid water, excluding natural precipitation and artificial irrigation [1,2]. NRW is an important component of the Land surface water balance; It includes of three soil components: soil distilled water,vascular water (or capillary water) and guttation. The liquid soil water in deeper layer rises to the surface due evaporates, and then condenses. This part of soil water is called the soil distilled water. The water in the soil pores has obvious surface tension due to the molecular gravity. The liquid water in the soil tends to migrate from the deeper to the surface by its own tension (potential) in the soil pores, forming the capillary suction phenomenon. It is called vascular water or capillary water.Guttation is the water-drop overflowing phenomenon through hydathodes of plant. It's caused by root pressure. It's classified as soil water because its origin water comes from the soil. Additionally, there are three atmospheric constituents: fog, dew and water-vapor adsorption (WVA) (Fig. 1). NRW has indeed been recognized as an important water source in arid and semi-arid environments [1,3]; additionally, it can greatly influence the ecological processes in arid and semi-arid areas [[4], [5], [6], [7], [8]]. However, at present, the soil components of NRW are almost unobserved, as the technology for observing them is not yet fully developed. At the current time, there is no direct observation instrument available to measure the atmosphere components of NRW, which can only be estimated indirectly from observation data; therefore, in the current study, NRW only refers to fog, dew and WVA [3,9].

The predominant instruments employed for observing NRW include a Hiltner Dew Balance [10,11], a leaf wetness sensor [12] and a microlysimeter or lysimeter [3,9]. The most reliable way to observe and measure NRW is by means of a lysimeter at present [13]; However, the accuracy of this data still needs to be improved.

A method in this paper will be established for identifying components of NRW, which is based on a scheme for identifying the components of NRW proposed by Zhang in 2011 and 2012 [14,15]. Clearly the identification process of NRW was given and the accuracy of the components of NRW was improved. The method is called A Quantitative Identification method for NRW(QINRW).The specific method is as follows:

## Observation of NRW

### Observation instruments

**Fig. 2:**
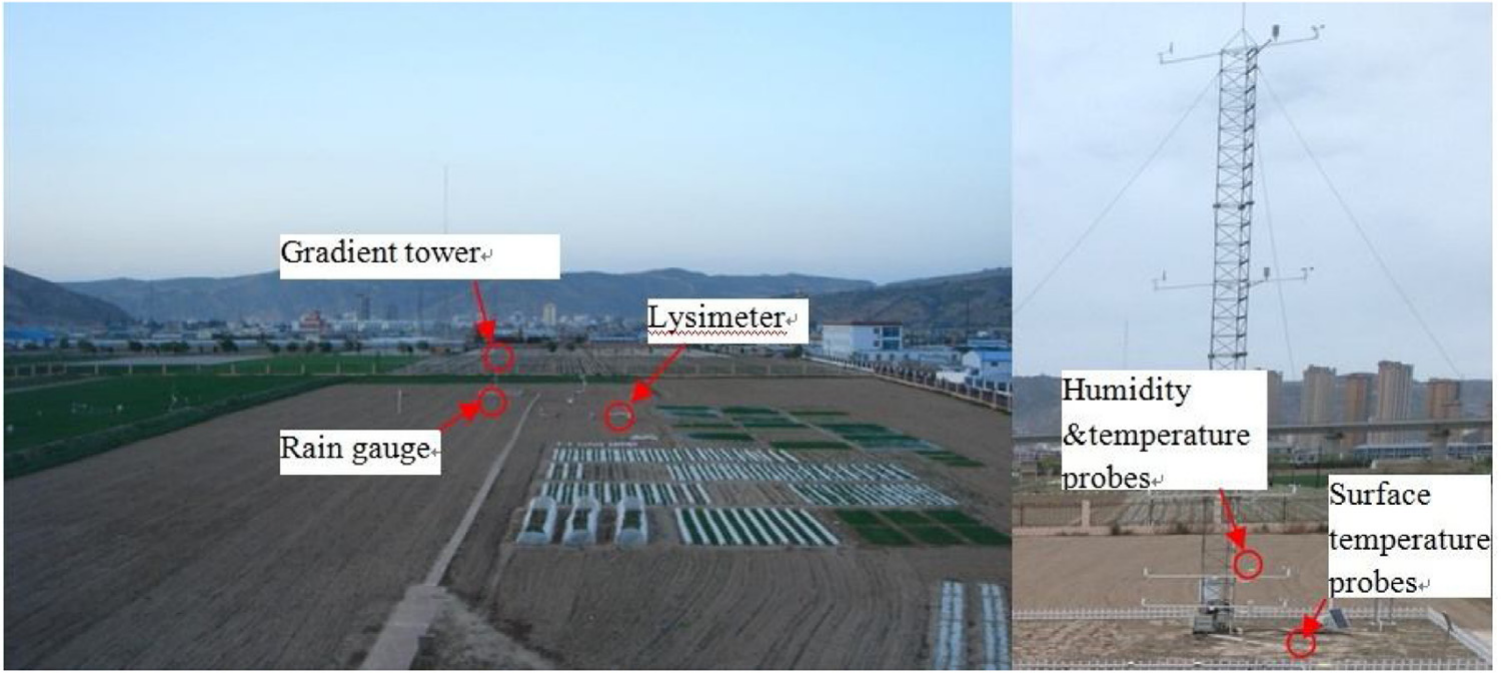
Environment of Dingxi station and gradient observation tower.

**Fig. 3:**
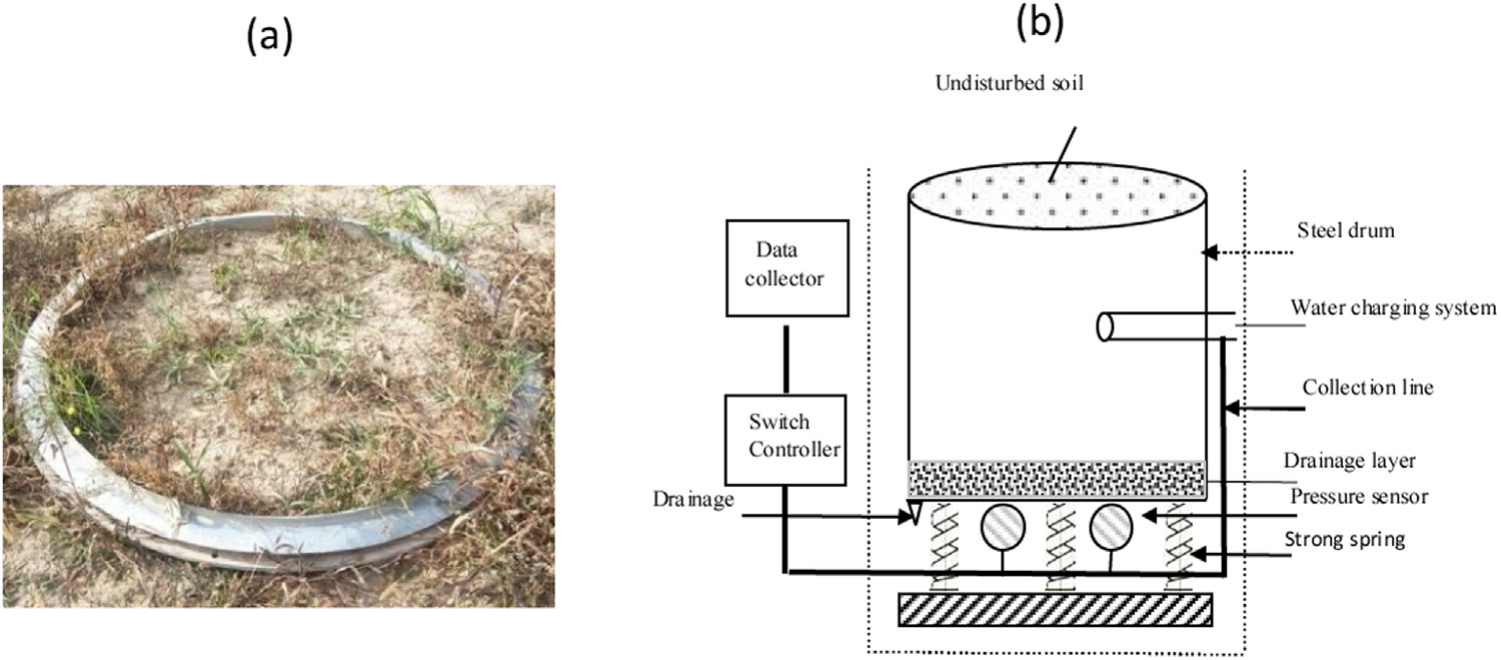
(a) Lysimeter in the field (b) Schematic of a weighing lysimeter.

The primary meteorological elements observed at Dingxi (Fig. 2) are wind, temperature and humidity gradients (1/2/4/10/16 m) with a sampling rate of every 10 min in the surface layer. We used HMP45D sensors to measure the air temperature and humidity (Fig. 2). The surface temperature (0 cm, model 109; Campbell Scientific, USA) with a sampling rate of every 10 min was also measured. Dust collectors are placed on the surface to collect dust on rainless days; the weight is then measured by an electronic balance. Land-surface actual evapotranspiration (ET) and NRW were measured with a large lysimeter (Fig. 3a) to an accuracy of 0.03 mm and a sensitivity of 0.01 mm. The High resolution sensors were used in the weighing system of the lysimeter, equipped with data collector and signal sensors; Its working principle is illustrated in Fig. 3b. When hourly changes in weight of the lysimeter data are below the accuracy, the data is considered low quality data. The diameter of the lysimer was 2.25 m and the effective evapotranspiration area was 4.0 m^2^ at a depth of 2.65 m; data was recorded every hour. Evapotranspiration during twilight and night [16,17], which can be considered to be an opposite water flux to NRW´s at night, naturally limit the occurrence of NRW. Using hourly values in our analyses, to a certain extent, might lead to an underestimation of NRW. The performance and accuracy parameters of the observational instruments are presented in Table 1.

**Table 1:** The experimental instruments and main technical parameters.

Instrument	Performance	Manufacturer	Height (m)
LG-I lysimeter	Accuracy: 0.03 mm; sensitivity: 0.01 mm	Institute of Arid Meteorology, China	Surface
HMP45D Humidity & Temperature probe	Temperature measurement range, −39.2– +60 °C with accuracy, ±0.2 °C; Relative humidity measuring range, 0.8–100 % with accuracy, ±1 %	Vaisala, Helsinki, Finland	1, 2, 4, 10 and 16
109 Temperature probe	Temperature Measuring range :-50–70 °C; accuracy:< ±0.2 °C	Campbell Scientific Inc., USA	0 cm on the soil
Rain gauge	Accuracy: ±2.5 %Volumetric Water Content(VWC) using standard calibration	Campbell Scientific Inc., USA	0.8 m above the soil
Dust collector (JJ1000 Electronic balance + Dust-full jar)	Accuracy: 0.01 g; Measuring range:0-1000g	Shuangjie Electronics Co., Ltd., China,	Surface and lab in Dingxi Station

### Lysimeter data pre-processed

Lysimeter data pre- and post-processing was needed to reduce the impact of noise on the determination of water balance components from lysimeter observations [[18], [19], [20], [21], [22], [23]]. The data filtered [18] was adopted in this paper. Firstly, the data during rainfall, snowfall and sandstorm periods were removed. Secondly, outliers during unreliable periods were eliminated by visual detection. Then, a threshold filter was implemented to remove the unrealistic data. The threshold value in this paper was set to 0.08 mm/h which was based on the literature results [24]. The data greater than 0.08 mm/h was eliminated. Finally, we extrapolated short-term missing data by the linear trend interpolation method: namely, when a quantity was once an hour or twice in two hours, the linear interpolation was adapted by using 2 adjacent points, on average, according to the linear changing trend of quantity. There is no imputation for long-term missing data. Others,evapotranspiration at night and early morning was underestimated was from the robust time scale. Hourly data might lead to an underestimated evapotranspiration and then lead to a certain extent to an underestimation of NRW.

## Quantitative identification system of NRW component

**Fig. 4:**
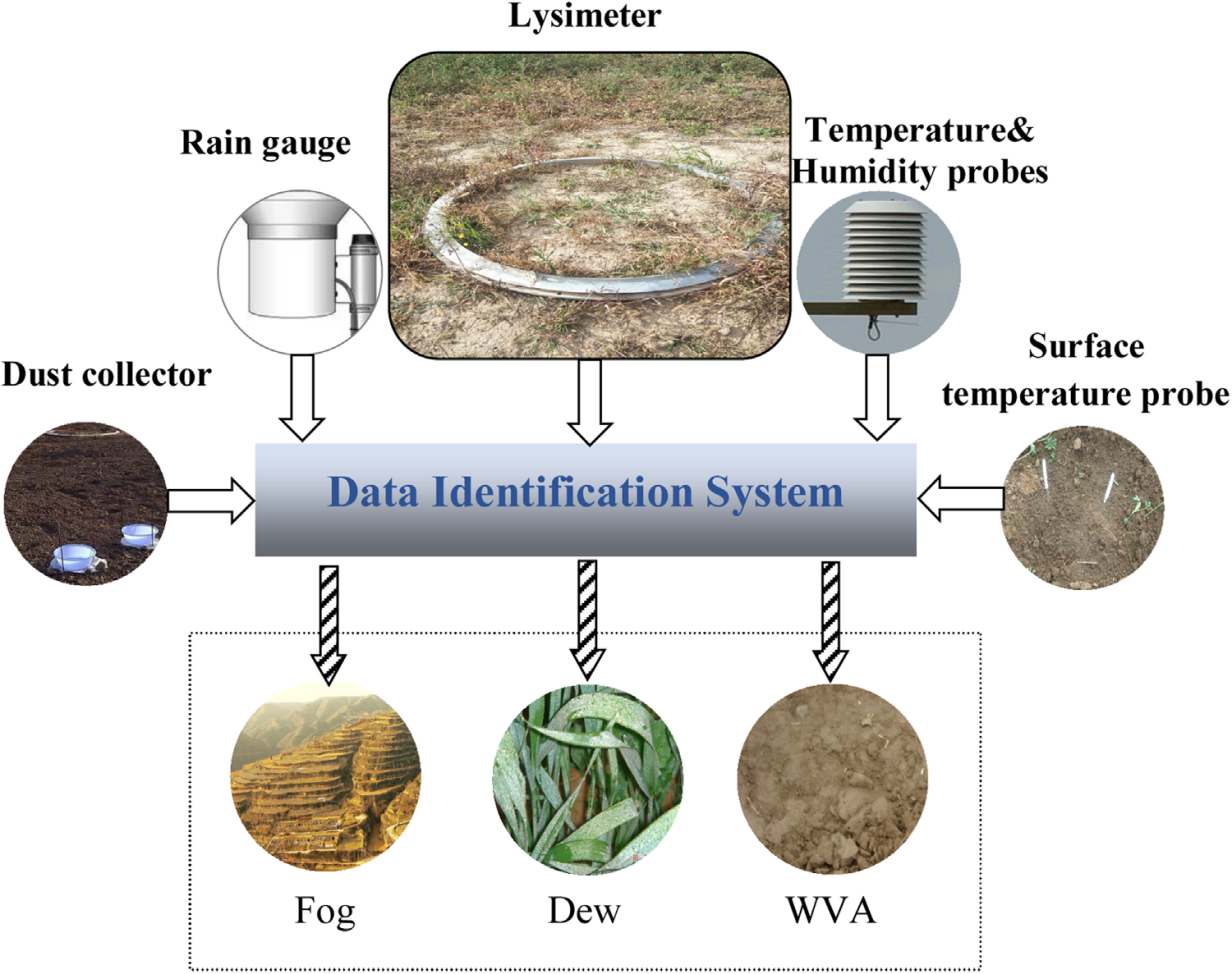
Quantitative identification system of NRW component.

The NRW component identification system in this paper is shown as Fig. 4. The system includes a data identification system and necessary observation instruments, including: lysimeter, rain gauge, dust deposition collector, air temperature and humidity sensor and surface temperature sensor. The above data were put into the data separation system; the output then derived from this system was the NRW component.

## Determination of NRW component of land surface water

**Fig. 5:**
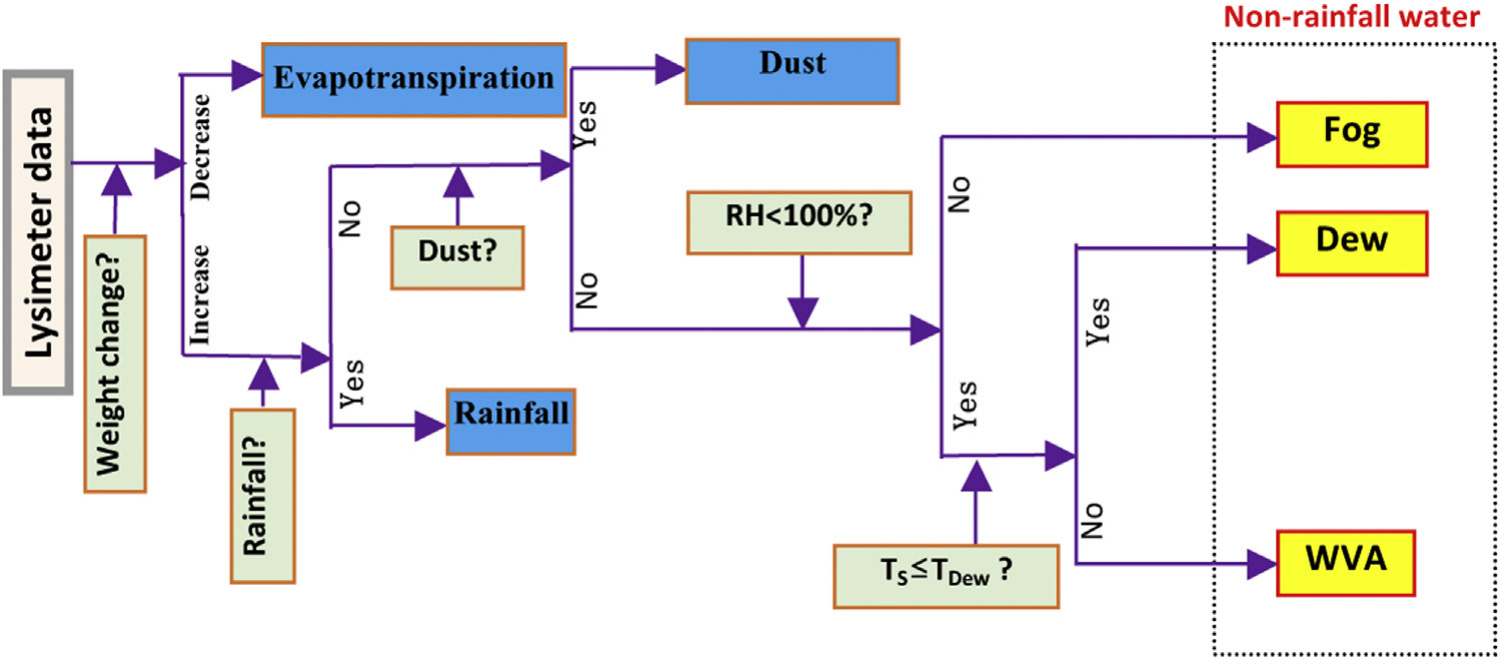
Data identification system to calculate NRW component (*ET* is evapotranspiration, *RH* is air relative humidity, *T*s is surface temperature, *T*_dew_ is dew-point temperature).

Previous studies only considered the effect of precipitation on the lysimeter data, but neglected the effect of dry deposition (dust). Due to the order of magnitude of NRW being very small, it will bring great error. Therefore, this needs to be combined with micro-meteorological and conventional meteorological observation data, in order to distinguish between different types of NRW observed with lysimeter weight changes. Herein, we use the method described in Fig. 5 to calculate the NRW.

To illustrate this more clearly, the variation of lysimeter data can be expressed as

(1) $$\Delta w=w_{i}-w_{i-1},$$

Where Δw is the variation value of the lysimeter [kg] (when there is no NRW, we have Δw = 0 or <0), *i* is the series number of the instantaneous value at each 60-min interval. *w_i_* and *w_i-1_* is weight value of number i and i-1by lysimeter [kg].

The dew-point temperature in this paper was calculated by a formula given by Michell Instruments Company. The formula is as follows:

On the water surface：

(2) $$T_{d}=\frac{243.12\times \ln(e_{w}/611.12)}{17.62-\ln(e_{w}/611.12)}$$

Where *T_d_* is dew-point temperature, e_w_ is saturated vapor pressure. The application scope of the formula is: −45 °C～+60 °C, the uncertainty of T_d_ in formula (2) is ±0.04 °C.

NRW components are obtained in five steps (Fig. 5) as follows :

a Firstly, the current data from the lysimeter is compared with the previous one. If Δw <0, it can be inferred that evapotranspiration happens in this time period, and no NRW is produced. On the contrary, if Δw >0, the value may be NRW.
b Secondly, it needs to be judged whether NRW occurs according to the precipitation data. On the basis of the first step, precipitation data are introduced. If precipitation occurs during the above observation period, it is assumed that the added value of lysimeter data is precipitation, not NRW; if no precipitation occurs, it is assumed that the added value may be NRW.
c Thirdly, NRW and dry deposition will be distinguished according to the dust data. Based on the second step, the dust data observed in the same period are imported. If there is a dust in the above observation period, it is considered that the reason for the increase of lysimeter data is sand dust; if no dust occurs, the increment value of lysimeter is determined as NRW.
d Next, in order to further determine the components of NRW, the air relative humidity data are introduced based on the last step. If the relative humidity of air is equal to 100 %, it is regarded as fog; otherwise it is considered as Dew or WVA.
e Finally, the surface temperature is used to distinguish dew and WVA. Comparing the surface temperature with the dew point temperature, if the surface temperature is lower than the dew point temperature, it is dew; otherwise, it is WVA.

Thus far, each component of NRW is determined.

## QINRW validation

**Fig. 6:**
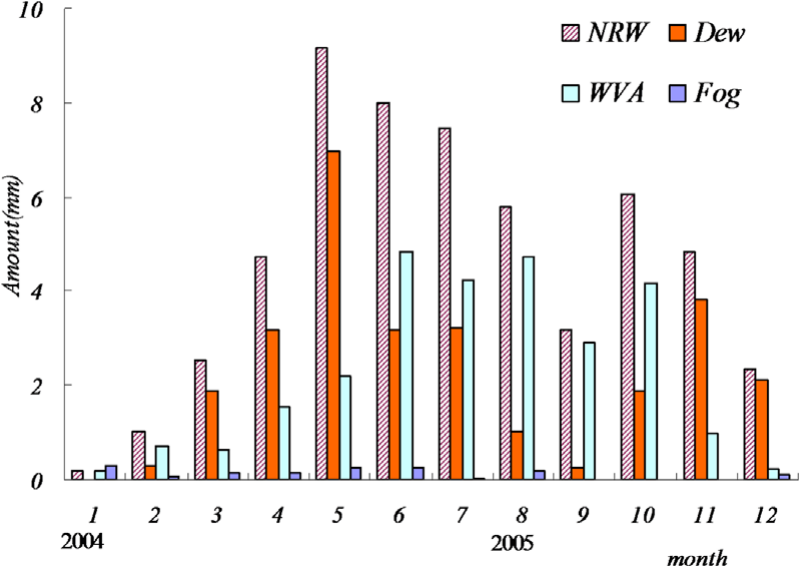
Distribution of NRW components in Dingxi station.

Data in Dingxi station of China is used to test our method (Fig.6). At present, there are almost no research results to show the variation characteristics of NRW throughout a whole year. Our findings show that the NRW in autumn is the most whereas in summer it is the least: this can be attributed to the summer temperature being high, which is more conducive to evapotranspiration, thus reducing the formation of NRW. With the arrival of autumn and the decreasing temperature, the increasing NRW amount is a reasonable result, a finding consistent with the literature [5]. Employed according to our method, Table 2 shows the sum of NRW components, represented as ten day periods, throughout a whole year.

**Table 2:** Amount of NRW component in each 10-day period in a whole year.

Year	Month	10-day period	Dew(mm)	WVA(mm)	Fog(mm)
2004	6	Early	0	0.01	0
		Mid-term	0	0.03	0
		Late	0	0.00	0.03
2004	7	Early	0.05	0.09	0.08
		Mid-term	0.11	0.47	0
		Late	0.16	0.07	0.01
2004	8	Early	0.46	0.11	0.04
		Mid-term	0.30	0.13	0
		Late	1.13	0.24	0.11
2004	9	Early	0.99	1.0	0.08
		Mid-term	1.67	0.38	0.09
		Late	0.51	0	0
2004	10	Early	1.69	0.62	0.07
		Mid-term	1.58	0.61	0.09
		Late	2.71	0.70	0.11
2004	11	Early	1.65	1.01	0.07
		Mid-term	0.33	2.24	0.01
		Late	0.95	1.31	0.20
2004	12	Early	2.18	2.42	0
		Mid-term	1.03	1.22	0.05
		Late	0	0.56	0
2005	1	Early	0.03	1.58	0.02
		Mid-term	0.65	0.96	0
		Late	0.36	2.02	0.17
2005	2	Early	0.03	0.98	0
		Mid-term	0	0.83	0
		Late	0.25	1.09	0
2005	3	Early	0.22	1.83	0
		Mid-term	0.44	1.14	0
		Late	1.23	1.22	0
2005	4	Early	2.74	0.82	0
		Mid-term	0.07	0.12	0
		Late	0.31	0.06	0
2005	5	Early	0.51	0	0
		Mid-term	0.24	0.07	0.04
		Late	1.38	0.04	0.06

Undoubtedly, the measurement of NRW is a challenging task as its order of magnitude is very small [12]. Therefore, in the process of quantitative identification of NRW, the influence of dust should be considered, especially in arid and semi-arid regions. In theory, so far QINRW is the most rigorous way to estimate NRW by using lysimeter data, although it requires more validation. Lastly, this method can be also applicable to the data from the microlysimeter.

## Declaration of competing interest

The authors declare that they have no known competing financial interests or personal relationships that could have appeared to influence the work reported in this paper.
